# Membrane Protein Location-Dependent Regulation by PI3K (III) and Rabenosyn-5 in *Drosophila* Wing Cells

**DOI:** 10.1371/journal.pone.0007306

**Published:** 2009-10-02

**Authors:** Masato Abe, Yuka Setoguchi, Tsubasa Tanaka, Wakae Awano, Kuniaki Takahashi, Ryu Ueda, Akira Nakamura, Satoshi Goto

**Affiliations:** 1 Glycobiology and Glycotechnology Research Group, Mitsubishi Kagaku Institute of Life Sciences, Tokyo, Japan; 2 Mutant Flies Laboratory, Mitsubishi Kagaku Institute of Life Sciences, Tokyo, Japan; 3 Laboratory for Germline Development, RIKEN Center for Development Biology, Hyogo, Japan; 4 Invertebrate Genetics Laboratory, National Institute of Genetics, Shizuoka, Japan; 5 Precursory Research for Embryonic Science and Technology (PRESTO), Japan Science and Technology Agency, Saitama, Japan; University of Texas MD Anderson Cancer Center, United States of America

## Abstract

The class III phosphatidylinositol-3 kinase (PI3K (III)) regulates intracellular vesicular transport at multiple steps through the production of phosphatidylinositol-3-phosphate (PI(3)P). While the localization of proteins at distinct membrane domains are likely regulated in different ways, the roles of PI3K (III) and its effectors have not been extensively investigated in a polarized cell during tissue development. In this study, we examined *in vivo* functions of PI3K (III) and its effector candidate Rabenosyn-5 (Rbsn-5) in *Drosophila* wing primordial cells, which are polarized along the apical-basal axis. Knockdown of the PI3K (III) subunit *Vps15* resulted in an accumulation of the apical junctional proteins *D*E-cadherin and Flamingo and also the basal membrane protein β-integrin in intracellular vesicles. By contrast, knockdown of PI3K (III) increased lateral membrane-localized Fasciclin III (Fas III). Importantly, loss-of-function mutation of *Rbsn-5* recapitulated the aberrant localization phenotypes of β-integrin and Fas III, but not those of *D*E-cadherin and Flamingo. These results suggest that PI3K (III) differentially regulates localization of proteins at distinct membrane domains and that Rbsn-5 mediates only a part of the PI3K (III)-dependent processes.

## Introduction

Cell polarity along the apical-basal axis is essential for the function of epithelial cells. This polarity is formed and maintained by distinct localization of membrane spanning and associated proteins, to apical, lateral or basal membrane domains. Membrane proteins localized to the apical or basolateral plasma membrane are endocytosed into early and apical or basolateral endosomes. For example, horseradish peroxidase (HRP) administered to the apical cell surface is incorporated into the apical early endosome. By contrast, HRP or dimeric IgA administered to the basolateral cell surface or transferring receptor (TfR) in the basolateral domain are internalized into the basolateral early endosome, which remain distinct [1]–[4]. Sorting of proteins for transcytosis, recycling and degradation takes place in these early endosomes. The proteins, incorporated into apical and basolateral early endosomes, meet in common endosomes, a process that can be observed within 15 min after the onset of internalization in MDCK cells [4]. The significance of keeping the apical and basolateral early endosomes distinct is thought to ensure that proteins from the apical and basolateral plasma membrane remain apart before the sorting processes proceeds. Although it is plausible that the trafficking of proteins in distinct membrane domains is regulated differently, the factors involved in such a differential regulation remain elusive.

One of the key molecules regulating membrane trafficking is PI3K (III), a heterodimer of Vps34p and Vps15p/p150, which produces phosphatidylinositol-3-phosphate (PI(3)P) [5]–[8]. PI(3)P is found to localize with early endosome and internal vesicles of multivesicular bodies (MVBs) in mammalian cells in culture [9]. Genetic and pharmacological analysis, using yeast and mammalian cells in culture, suggests that PI3K (III) is required for five distinct processes. These are: (i) the fusion of clathrin-coated vesicles and early endosomes as well as the fusion between early endosomes [10]–[13]; (ii) the recycling from early endosomes back to the Golgi complex or other destinations [14], [15]; (iii) the entry of proteins into the lysosomal degradation pathway [16]–[22]; (iv) the formation of internal vesicles of MVBs [23], [24] and (v) autophagy [25], [26]. Moreover, inactivation of PI3K (III) by *Vps34* mutation leads to an expansion of the outer nuclear membrane and an abnormal reduction of the LDL receptor at the apical membrane in *C. elegans* [27]. In *Drosophila*, *dVps34* mutation results in defective endocytosis of the apical membrane protein Notch and a defective onset of autophagy [28]. It has been suggested that PI3K (III) utilizes different effectors at apical and basolateral endosomes [15]. However, the role of PI3K (III) in the regulation of protein localization at different membrane domains has remained unclear.

To understand the various functions of PI3K (III), it is crucial to clarify which downstream effectors are involved in each of the processes it regulates. PI3K (III) is thought to exert its function through the recruitment of proteins that contain PI(3)P-binding motifs such as FYVE or PX domains [29], [30]. Among such proteins, Rabenosyn-5 (Rbsn-5) has been shown to contribute to endosome fusion and recycling processes in mammalian cells [31]–[33]. Genetic studies on *C. elegans* and *Drosophila* also show that Rbsn-5 is essential for receptor-mediated endocytosis and endosome fusion [34], [35], although it is not clear whether or not Rbsn-5 is involved in other PI3K (III)-related phenomena.

To determine how the proteins in distinct membrane domains are regulated by PI3K (III) and its effector Rbsn-5 we analyzed *Drosophila* wing development. This provides a good model since wing primordial cells have a clear polarity along the apical-basal axis [36]. In addition a number of membrane proteins are known to be transported in an organized manner along the apical-basal axis. For example *D*E-cadherin, a cell adhesion protein and Fmi, a planar cell polarity (PCP) core protein, are localized in the apical junctions or zonula adherens (ZA) [36], [37], whereas the cell adhesion molecules FasIII and β-integrin are localized in lateral and basal membranes, respectively [38], [39]. In this study, it was found that inactivation of PI3K (III) in the wing primordial cells by knockdown of *dVps15* affects the localization of these membrane proteins differently. In particular, we found that *dVps15* knockdown resulted in the accumulation of FasIII at the lateral membrane, whereas it resulted in intracellular accumulation of *D*E-cadherin, Fmi and β-integrin. Importantly, inactivation of *Rbsn-5* showed accumulation of FasIII and β-integrin at the lateral membrane and intracellular vesicles, respectively, but no effects of *D*E-cadherin and Fmi localization. These results provide evidence for a differential regulation of protein localization by PI3K (III) and Rbsn-5 at distinct membrane domains.

## Results

### Gal4/UAS -mediated knockdown of PI3K (III) in *Drosophila*

**Figure 1:**
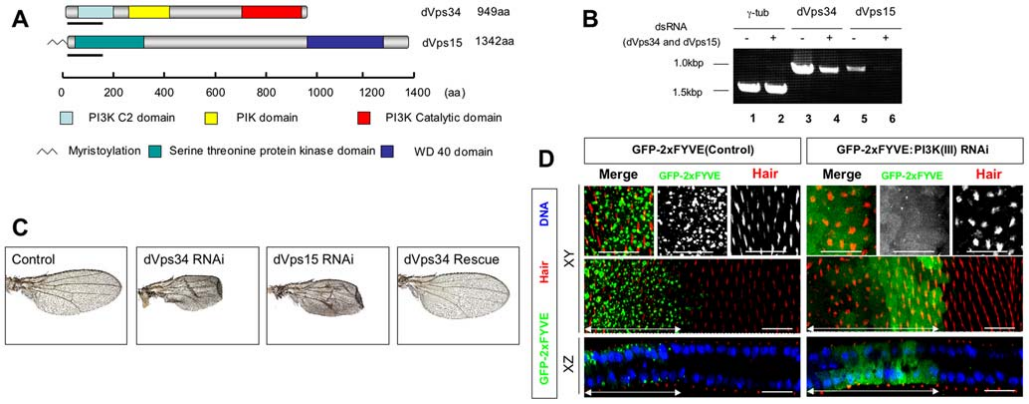
Gal4/UAS mediated RNA interference in *Drosophila.* (A) Schematic representation of *Drosophila* phosphatidylinosytol 3 kinase class III (PI3K(III)) composed of dVps34 (a catalytic subunit, CG5373) and dVps15 (an adaptor subunit, CG9746). PI3K C2 domain, PI kinase conserved domain (PIK domain), PI3K catalytic domain, serine threonine protein kinase domain, WD 40 domain and N-terminal myristoylation are indicated. Underlining indicates regions used to construct dsRNA inducible vectors. (B) dVps34 and dVps15 mRNA abundance were reduced by transfection of the respective dsRNA (+) in S2 cells. RNAs was quantified by RT-PCR. γ-tubulin was used as a control. (C) Adult wings in which dsRNAs for either *dVps34* (dVps34 RNAi) or *dVps15* (dVps15 RNAi) were expressed by the *sd*-Gal4 driver. The severe malformation was completely rescued by co-expression of wild-type cDNA with dsRNA for *dVps34* (dVps34 Rescue), compared with the wild-type wing (Control). (D) Confocal immunodetection showing XY and XZ sections in the plane of pupal wings at 32 h APF. Double-headed arrows indicate the regions where only GFP-2xFYVE, or both GFP-2xFYVE and dsRNA for *dVps15*, were expressed using the *dpp*-Gal4 driver. GFP-2xFYVE (green) was localized to dot-like structures in the wild type but dispersed in the cytoplasm in the *dVps15*-knockdown cells. Wing hairs stained by rhodamine phalloidin (red) were pointed and orderly in the wild type but deformed and irregularly pointed in the *dVps15*-knockdown cells. The scale bars represent 10 µm.

*Drosophila* PI3K (III) consists of two essential components, the catalytic subunit dVps34/PI3K59F (CG5373) and the anchoring subunit dVps15/p150 (CG9746 (see Fig. 1A). To analyze the function of PI3K (III), transgenic fly strains were generated harboring ∼500 bp of inverted cDNA fragment repeats, corresponding to the PI3K (III) subunits, under the control of the GAL4-responsive UAS. Crossing these strains with appropriate Gal4 driver strains induces a hairpin type double strand RNA (dsRNA) in a Gal4-dependent manner, both temporally and spatially. Accordingly, dsRNAs in whole or restricted regions of the wing primordia were induced using *scalloped*-Gal4 (*sd*-Gal4) or *decapentaplegic*-Gal4 (*dpp*-Gal4) driver strains, respectively.

The knockdown of either *dVps34* or *dVps15* by *sd*-Gal4 resulted in a severe malformation of the wings (Fig. 1C), with similar phenotypes being induced by a knockdown of either *dVps34* or *dVps15*. To test whether PI3K (III) is responsible for this phenotype, we performed a rescue experiment by examining whether the expression of the wild-type cDNA would suppress the phenotype induced by the dsRNA-mediated knockdown. It was found that the defects induced by knockdown of *dVps34* were completely suppressed when the wild-type cDNA of *dVps34* was simultaneously expressed (Fig. 1C).

To examine further the dsRNA-mediated knockdown reduction of *in vivo* activity of the PI3K (III), the localization of tandem-repeated FYVE domains fused to GFP (GFP-2xFYVE) was investigated [40] (Fig. 1D). GFP-2xFYVE is localized with PI(3)P-rich membrane domains in endosomes, since the FYVE domain binds to PI(3)P generated by PI3K (III) (Fig. 1D). When we reduced *dVps15* expression in the wing cells by inducing *dVps15* dsRNA, GFP-2xFYVE was no longer localized to the endosomes, but rather was dispersed within the cytoplasm (Fig. 1D), suggesting that the knockdown significantly reduced PI3K (III) activity in *Drosophila*. The above results taken together led us to conclude that the defects were caused by a reduction in the level of PI3K (III) activity.

### PI3K (III) is required for efficient endosomal trafficking to the lysosomes

**Figure 2:**
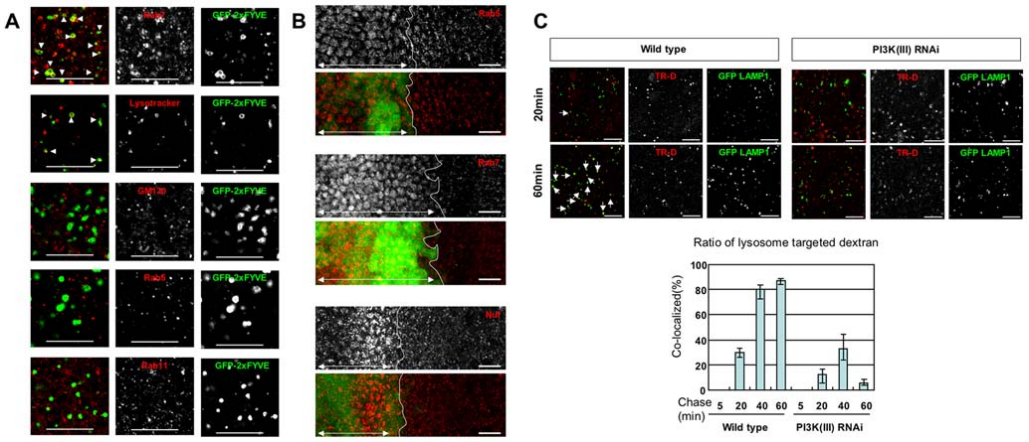
Endocytotic trafficking is compromised by knockdown of PI3K (III). (A) GFP-2xFYVE co-localized with Rab7 and Lysotracker but not with GM130, Rab5 or Rab11. Arrowheads point to the GFP-2xFYVE-positive compartments with Rab7 and Lysotracker. (B) The numbers of Rab5-, Rab7- and Nuf-positive endosomes (red) were increased in the *dVps15*-knockdown cells (left sides of white lines, indicated by double-headed arrows) compared with the wild type cells (right sides of white lines). The GFP-2xFYVE (green) and dsRNA for *dVps15* were simultaneously expressed by *dpp*-Gal4 driver. (C) *Upper panels*: Trafficking of TR-D from the plasma membrane to the lysosome was monitored by double labeling of TR-D and GFP-LAMP1 (lysosome marker, green) in the wing discs. Wing discs of the wild type or *dVps15* knockdown were pulse labeled with TR-D for 5 min and chased in Schneider's medium for the indicated times. The wing discs were then fixed and analyzed by confocal microscopy. Arrows show the co-localization of TR-D and GFP- LAMP1. The scale bars represent 10 µm. *Lower graph*: The ratios of co-localized TR-D to GFP-LAMP1 in the wild or *dVps15* knockdown discs (*n* = 89∼152).

The Rab family of GTPases plays pivotal roles in the regulation of vesicular trafficking, the Rab5, Rab7 and Rab11 members contributing to the formation and dynamics of early endosomes, late endosomes and recycling endosomes, respectively [41]. It seems likely that PI3K (III) products might co-localize with these Rab family proteins since they are known to physically associate with Rab5 [10], and possibly with Rab7 [42]. Indeed, GFP-2xFYVE has been shown to localize with Rab5-positive endosomes, when expressed in *Drosophila* S2 and neural cells [40]. We therefore examined which endosomal compartments GFP-2xFYVE is localized to when expressed in larval wing cells. In larval wing disc cells GFP-2xFYVE was localized mostly to Rab7-positive endosomes, but not to Rab5- or Rab11-positive endosomes (Fig. 2A). In addition, some of the GFP-2xFYVE-positive endosomes were stained with Lysotracker (Fig. 2A), suggesting that they were acidic, including the late endosomes. PI3K (III) products therefore appear to localize to Rab7-positive late endosomes in these cells.

Although PI3K (III) has been implicated in the fusion of clathrin-coated vesicles and early endosomes and also in the fusion between early endosomes, it has been unclear whether reduced PI3K (III) activity impairs these endosomes in *Drosophila* wing disc cells. Knockdown of *dVps15* markedly increased the numbers of Rab5-positive and Rab7-positive dot-like structures, as shown in Fig. 2B, suggesting that the inactivation of PI3K (III) leads to the accumulation of early and late endosomes. Interestingly, knockdown of *dVps15* also increased the numbers of endosomes positive for the Rab11 effector Nuf [43], [44], indicating that PI3K (III) plays either a direct or an indirect role in the regulation of recycling endosomes. The abnormal accumulation of endosomal markers raises the possibility that PI3K (III) is required for the endosomal trafficking and endosome formation.

The question of whether the inactivation of PI3K (III) also affects the vesicular trafficking leading to lysosomes in *Drosophila* wing cells was then examined, since it has been demonstrated with other cell types [20], [28]. To this end, the incorporation and intracellular trafficking efficiencies of Texas Red-labeled dextran (TR-D) in the wing discs was determined. Discs from wild type and knockdown larvae expressing GFP-LAMP1, a lysosomal marker [45], were placed in Schneider's medium containing TR-D for 5 min and then washed. At 5, 20, 40, or 60 min after washing, discs were fixed and the number of TR-D-positive lysosomes counted (Fig. 2C). At all time points observed the percentage of GFP-LAMP1 and TR-D double-positive vesicles (TR-D-positive lysosomes) among the TR-D positive vesicles was clearly lower in the knockdown cells, compared with the normal cells (Fig. 2C). This suggests that intracellular endosomal trafficking to the lysosome is impaired by knockdown of PI3K (III).

### PI3K (III) is required for lysosome maturation

**Figure 3:**
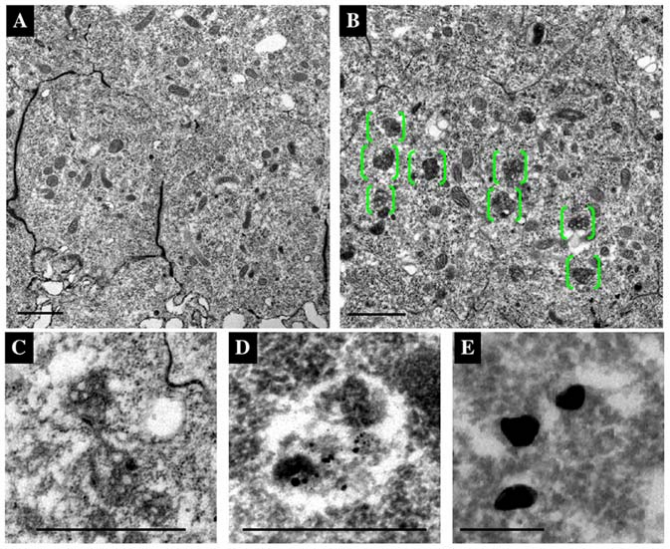
Abnormal MVB-like structures were observed in PI3K (III)-knockdown pupal wing cells. (A–E) Horizontal EM images of the pupal wings of the wild type (A and E), or *dVps15* knockdown (B–D), at 30 h APF. A number of electron-dense compartments appeared in the cytoplasm at the apical side (green parentheses in B). These electron-dense compartments appeared to be MVB-like structures (C and D) that were scarcely observed in the wild type cells (A). (D and E) Immuno-EM images of the pupal wings obtained with a 10 nm gold particle-labeled GFP antibody to detect GFP- LAMP1. Signals of anti-GFP antibody in the wild type (E) and *dVps15*-knockdown wings (D) are shown, respectively. The scale bars represent 1 µm (A–C), 500 nm (D) and 200 nm (E), respectively.

Lysosomes mature from MVBs, and PI3K (III) is necessary for the formation of internal vesicles within the MVBs in mammalian cells. We therefore examined whether maturation of MVBs also requires PI3K (III) activity in *Drosophila*. Knockdown cells were observed at high resolution using a TEM (transmission electron microscopy), and it was found that the number of abnormal MVB-like structures markedly increased (Fig. 3B and C), although these compartments were rarely observed in wild type cells (Fig. 3A). These abnormal MVB-like structures were preferentially localized within the apical region but not in the basolateral region of the knockdown cells (data not shown). Also the MVB-like structures were GFP-LAMP1-positive (Fig. 3D), suggesting that they may be immature lysosomes. It can therefore be concluded that PI3K (III) is required for the maturation of MVBs into lysosomes in the apical region of the cells.

### PI3K (III) regulates the localization of Fmi and *D*E-cadherin

**Figure 4:**
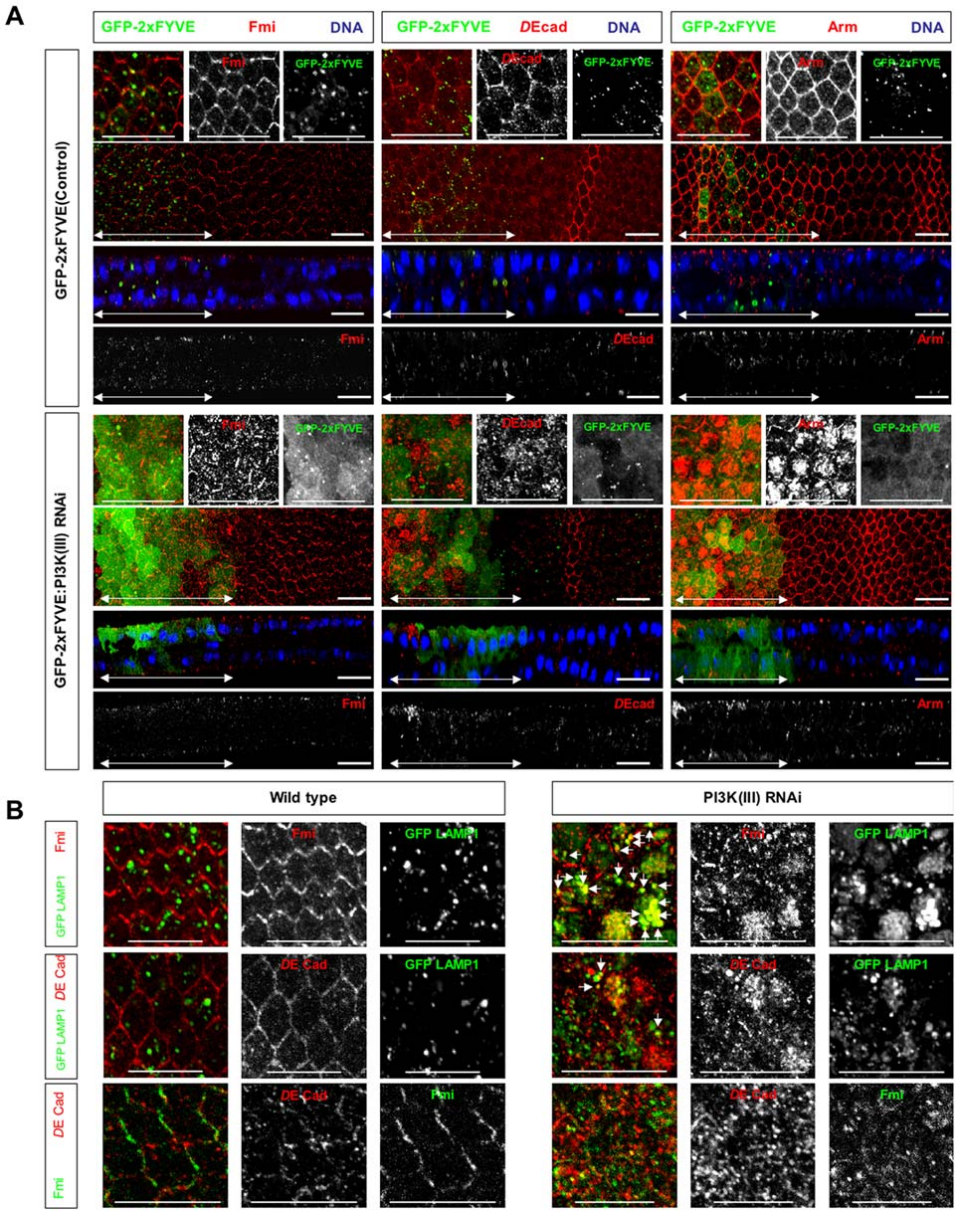
Fmi, *D*E-cadherin and Arm show intracellular accumulation in PI3K (III)-knockdown pupal wings. (A) Confocal immunodetection showing XY and XZ sections in the plane of pupal wings at 30–32hr APF. Flamingo [Fmi], *D*E-cadherin [*D*E-cad] and Armadillo [Arm] (red) were localized to the ZA in the plasma membrane in wild type, but accumulated to the intracellular puncta in *dVps15*-knockdown cells. The regions where the GFP-2xFYVE and/or dsRNA for *dVps15* were expressed by the *dpp*-Gal4 driver are indicated by double-headed arrows. (B) Fmi mostly accumulated to GFP-LAMP1 positive compartments in *dVps15*-knockdown pupal wing cells at 30 hr APF while *D*E-cadherin co-localized only partly. Arrows indicate GFP-LAMP1 positive compartments that harbor Fmi or *D*E-cadherin. Fmi and *D*E-cadherin location showed little overlap. The scale bars represent 10 µm.

The above results on the regulation of lysosomal degradation processes are largely consistent with the previously reported functions of PI3K (III). We next analyzed the function of PI3K (III) in proteins that localize to distinct membrane domains along the apical-basal axis. In the apical membrane domain, Fmi, a planar cell polarity (PCP) protein, is localized to the apical side of the ZA. Fmi first localizes uniformly to the apical ZA and then accumulates in both the proximal and distal sides of the cell ∼30 h after pupal formation (APF) [37] (Fig. 4A). In *dVps15* knockdown cells, Fmi accumulated in intracellular small compartments in the apical region (Fig. 4A). The accumulated Fmi was found mostly in LAMP1-positive vesicles (Fig. 4B). Considering that PI3K (III) contributes to the maturation of MVBs into lysosomes, this result suggests that the degradation of Fmi is impaired by the *dVps15* knockdown.

*D*E-cadherin is known to localize to the ZA and in wild-type cells was localized mostly to the ZA and to some extent to other intracellular small compartments (Fig. 4A). However, knockdown of *dVps15* disrupted the localization of *D*E-cadherin at the ZA and a significant amount accumulated in the intracellular small compartments in the apical region (Fig. 4A). The accumulated *D*E-cadherin was partly localized to the LAMP1-positive compartments (Fig. 4B), suggesting that it was due, at least in part, to a defect in the degradation pathway. Finally, it was found that the compartments where *D*E-cadherin or Fmi accumulated showed little overlap (Fig. 4B). We also stained *Vps15*-knockdown discs with antibodies against *D*E-cadherin together with those against several compartment markers such as Rab5, Rab7 and Rab11. *D*E-cad co-localized partially with Rab11, but not with Rab5 or Rab7 (data not shown), suggesting that a fraction of *D*E-cad accumulates in recycling endosomes. The remainder of the *D*E-cad was localized in unidentified compartments which were negative for both of these markers.

*D*E-cadherin is known to bind to its intracellular partner Arm, accordingly whether the localization of Arm is also affected by the knockdown of PI3K (III) was investigated. In wild type cells, Arm was localized to the ZA (Fig. 4A). However, in the *dVps15* knockdown region, Arm accumulated in the apical small compartment (Fig. 4A), similar to *D*E-cadherin. Together, these results suggest that PI3K (III) is required for the proper localization of Fmi and the *D*E-cadherin-Arm complex to the ZA.

### PI3K (III) regulates the localization of Fas III and β-integrin in a distinct manner

**Figure 5:**
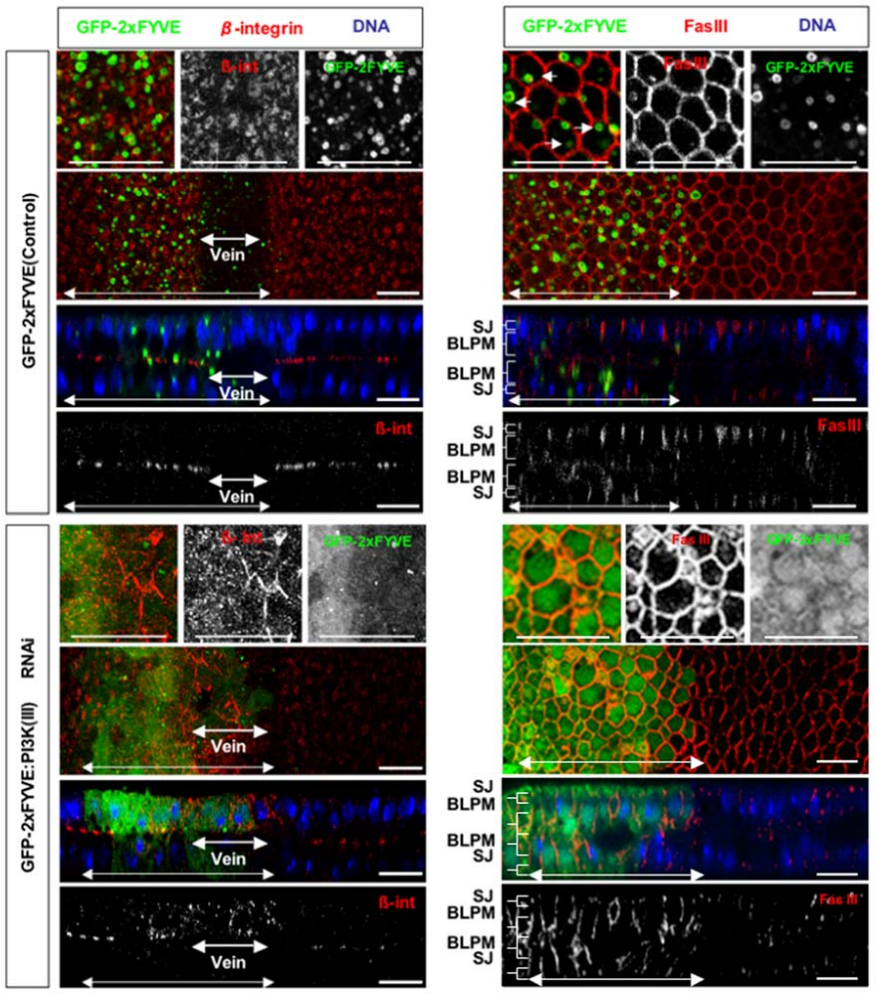
Subcellular localization defects of β-integrin and Fas III in PI3K (III)-knockdown pupal wings. Confocal immunodetection showing XY and XZ sections in the plane of pupal wings at 30 h APF. Double-headed arrows indicate the regions where the dsRNA for *dVps15* was expressed by dpp-Gal4 driver. β-integrin [β-int] (red) was localized to the basal plasma membrane in the wild-type prospective intervein regions but was only rarely localized to the basal plasma membrane while it accumulated in the cytoplasm in *dVps15*-knockdown cells. β-integrin was not degraded in the knockdown vein region neither was it observed in the wild-type vein region. By contrast, Fas III (red) was localized only to the SJ (indicated by brackets) in the wild-type while in the *dVps15*-knockdown cells it was accumulated to both the SJ and BLPM regions. The scale bars represent 10 µm.

It was next determined whether PI3K (III) also regulates the localization of basolateral membrane proteins. β-integrin was localized in intracellular vesicles at early pupal stages, but gradually translocated and accumulated to the basal junctions (data not shown). At 30 h APF, β-integrin formed large basal junctions at the basal plasma membrane of the prospective intervein cells (Fig. 5). By contrast, when *dVps15* was knocked down, β-integrin remained dispersed in the cytoplasm with small intracellular vesicles, and it failed to form basal junctions in the intervein region, even at 30 h APF (Fig. 5). This suggests that PI3K (III) might contribute either to the translocation of β-integrin to the basal membrane, or to the suppression of endocytosis of β-integrin, or both.

The β-integrin disappeared in prospective vein cells by 30 h APF since they are devoid of basal junctions (Fig. 5). Interestingly, β-integrin clearly accumulated at the intracellular vesicles in the cytoplasm, even in the prospective vein region (Fig. 5), suggesting that its degradation of might be compromised by the knockdown of *dVps15*.

Fas III is present at high levels at the septate junction (SJ), lateral cell junction, and at low levels in the remaining lateral membrane domain [39]. Fas III is first localized to both the SJ and more basal regions where Discs Large (Dlg), another component of the SJ, is absent. By 50 h APF, most of the adhesion structures in the lateral plasma membrane apparently disappear [38], probably because Fas III becomes delocalized from the lateral plasma membrane and is degraded by this stage. Here we examined the effect of PI3K (III) on the localization of Fas III at 30 h APF and it mostly disappeared from the basal region of the lateral plasma membrane but remained in the SJ (Fig. 5). The *dVps15* knockdown greatly increased the amount of Fas III remaining in the lateral plasma membrane region (BLPM, Fig. 5) even at 30 h APF. This phenotype is strikingly distinct from that of the intracellular accumulation of Fmi, *D*E-cadherin and β-integrin induced by *dVps15* knockdown. These results suggest that PI3K (III) might regulate Fas III and these other proteins in a different manner.

### Rbsn-5, a FYVE domain-containing protein, is selectively required for the proper localization of Fas III and β-integrin

**Figure 6:**
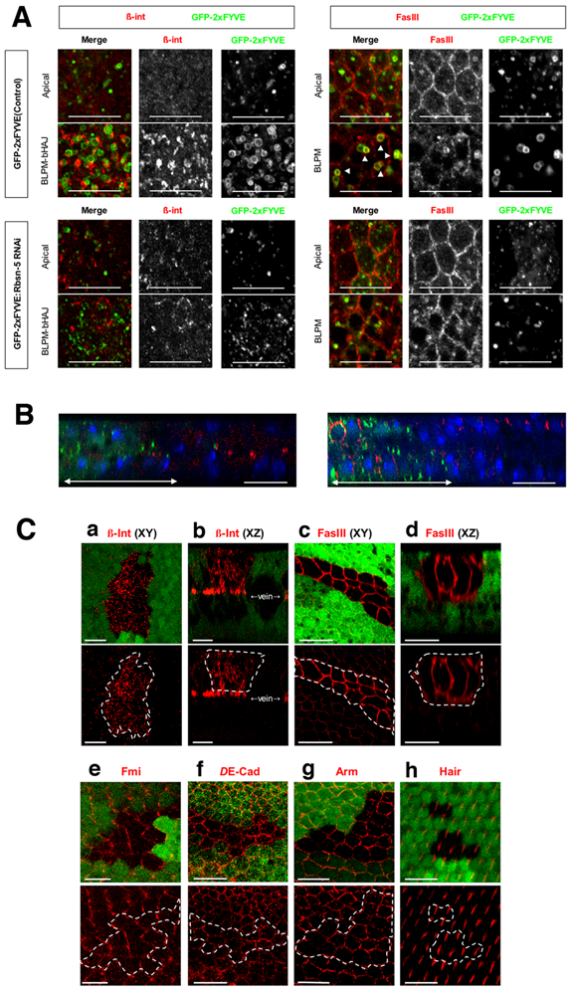
Rbsn-5 was selectively required for the correct localization of FasIII and β-integrin. (A) Horizontal sections of 27 h APF pupal wings, expressing GFP-2xFYVE, with or without dsRNA, for *Rbsn-5* (lower or upper panels, respectively). Images were obtained for repeated 1 µm sections from the apical side. Merged horizontal sections in the plane of the ZA and septate junctions are shown as Apical and those in the plane between the BLPM and bHAJ are indicated as BLPM-bHAJ. β-integrin was mostly localized to the basal plasma membrane and formed large clusters. In *Rbsn-5* knockdown cells, β-integrin was rarely localized to the basal plasma membrane and there was only a small amount of accumulation in the apical region. Fas III was localized to the SJ, whereas in *Rbsn-5* knockdown cells it also accumulated in the BLPM. In addition, two types of GFP-2xFYVE-positive endosomes were observed as dot-like small structures in the apical regions and vesicle-like large structures in basal regions in the wild type. In *Rbsn-5* knockdown cells, very few large endosomes were observed in the basal regions. Note that the large endosomes were very close to the BLPM membrane and frequently contained Fas III. (B) Vertical sections of the pupal wings expressing GFP-2xFYVE (green) with dsRNA for *Rbsn-5*. (left) β-integrin (red) was not localized to the basal junctions in the knockdown cells where the GFP-2xFYVE is expressed (green). (right) Localization of Fas III (red) extended to the BLPM in the knockdown cells (green). The normal distributions of these proteins were presented in the internal control regions where GFP-2xFYVE was not expressed. (C) Confocal immunodetection showing XY (a, c, e, f, g and h) and XZ (b and d) sections in the plane of the pupal wings at 30–32 hr APF. Cells that are encircled by white lines were GFP-negative and mutant for *Rbsn-5^C241^*. β-integrin (red in a and b) accumulated intracellularly, as compared with the wild-type cells surrounding the mutant cells. Fas III (red in c and d) accumulated in the whole basolateral plasma membrane, as compared with the GFP-positive wild-type cells. Fmi (red in e), *D*E-cadherin (red in f), Arm (red in g) and wing hairs (red in h) were normal. The scale bars represent 10 µm.

The above results indicate that PI3K (III) is required for the degradation and correct localization of Fmi, *D*E-cadherin, Fas III and β-integrin. To identify the factor under the control of PI(3)P, we selected genes from the *Drosophila* genome database that contain FYVE and/or PX domains, since these domains are known to bind to PI(3)P. The dsRNAs were then expressed, corresponding to each of the selected genes in the wing discs, and the effect on the localization of these membrane proteins determined. From this screen, we found that a *Drosophila Rbsn-5* knockdown influenced the localization of Fas III and β-integrin. In wild type cells, β-integrin was found to localize at or near the basal membrane as large clusters but was absent from the apical region at 27 h APF (Fig. 6A). However, knockdown of *Rbsn-5* markedly reduced the amount of β-integrin localized at or near the basal membrane and slightly increased the amount of intracellular vesicles located in the apical region (Fig. 6A and B). Furthermore, at 30 h APF for wild type cells, Fas III was mainly localized in the apical region of the lateral plasma membrane, while only a small fraction of Fas III was localized in the basolateral region (Fig. 6A). By contrast, when *Rbsn-5* was knocked down, Fas III was also found at the basal region of the lateral plasma membrane (Fig. 6A and B). These *Rbsn-5* knockdown cell phenotypes were reminiscent of those of the PI3K (III) knockdown cells.

**Figure 7:**
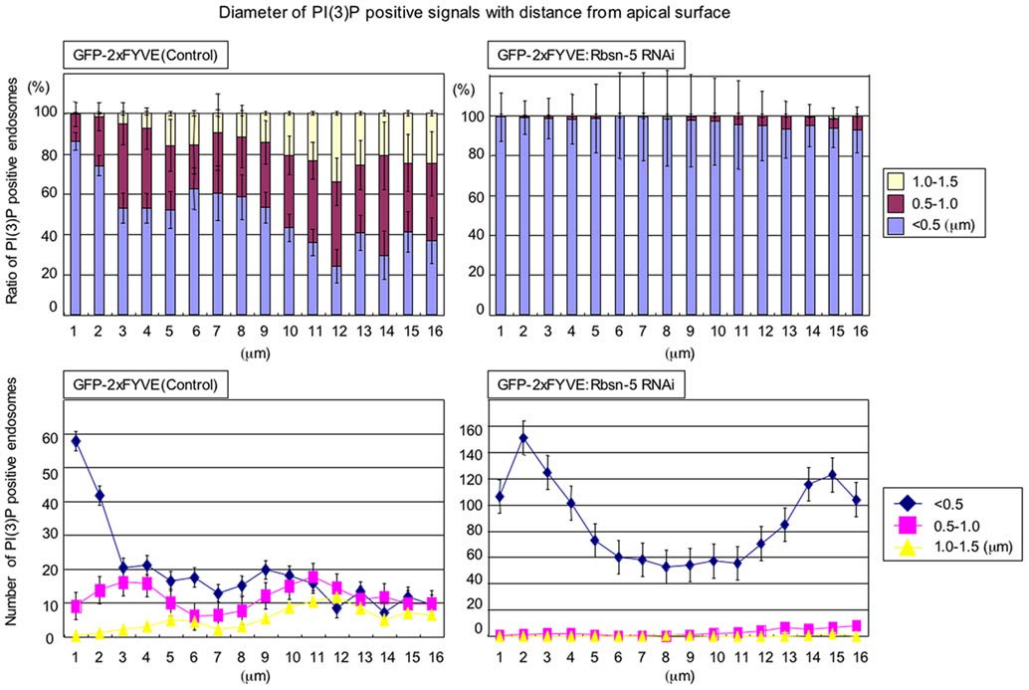
The number of basolateral large PI(3)P-positive endosomes was decreased in *Rbsn-5* knockdown pupal wings. Ratios (upper) or numbers (lower) of small (<0.5 µm, blue), middle (0.5–1.0 µm, red) and large (1.0–1.5 µm, yellow) endosomes are shown for repeated 1 µm sections from the apical to basal region in the wild type (left), or *Rbsn-5*-knockdown (right) wings.

To further investigate the roles of Rbsn-5 in the regulation of membrane protein localization, mutant cells were analyzed from a null allele for *Rbsn-5^C241^* [46]. Compared to the wild type cells (GFP-positive cells), β-integrin accumulated in significant amounts in the intracellular vesicles in the *rbsn-5* mutant clone cells (GFP-negative cells) at 30 h APF (Fig. 7C; a and b). In the case of Fas III, the amount that was localized to the basolateral plasma membrane was greatly increased (Fig. 7C: c and d). However, importantly, the *Rbsn-5* mutation did not result in an intracellular accumulation of Fmi, *D*E-cadherin or Arm, under the same conditions where it affected the localization of β-integrin and Fas III (Fig. 7C; e-g). Also the *Rbsn-5* mutation had no effect on the actin bundling that could be observed at the apical surface (Fig. 7C: h). Therefore, Rbsn-5 appears to selectively regulate the localization of β-integrin and Fas III in this context. These results strongly suggest that Rbsn-5 mediates only a part of the function of PI3K (III).

### Rbsn-5 increases the size of PI(3)P-positive endosomes, a phenomena prominent at the basal side of the cell

Given that Rbsn-5 regulates the localization of basolateral membrane proteins but not that of apical junctional membrane proteins, it is possible that it preferentially regulates basolateral vesicular trafficking. It has been reported in mammalian cells (WIF-B hepatocytes) that PI(3)P-positive endosomes at the apical regions are larger, relative to those at the basolateral region [15], however, with *Drosophila* wing disc cells the opposite was true. When PI(3)P-containing endosomes were visualized by GFP-2xFYVE, small endosomes (<0.5 µm diameter) were found more frequently at the apical side, whereas large endosomes (1.0–1.5 µm diameter) were found more frequently at the basal side in wild type cells (Fig. 6 and 7). Strikingly, the *Rbsn-5* knockdown reduced the size of GFP-2xFYVE-positive endosomes throughout the apical-basal axis of the cell (Fig. 6 and 7). This suggests that Rbsn-5 increases the size of PI(3)P-containing endosomes which appear more prominent in the basolateral regions, likely due to the fusion of small PI(3)P-containing endosomes.

The selective roles of Rbsn-5 in the regulation of basolateral membrane proteins prompted us to examine the localization of endogenous Rbsn-5. We found that Rbsn-5 was localized to intracellular endosomes at both the apical and basolateral regions (data not shown). It is therefore unlikely that the selective roles of Rbsn-5 were due to a selective localization of this molecule along the apical-basal axis.

**Figure 8:**
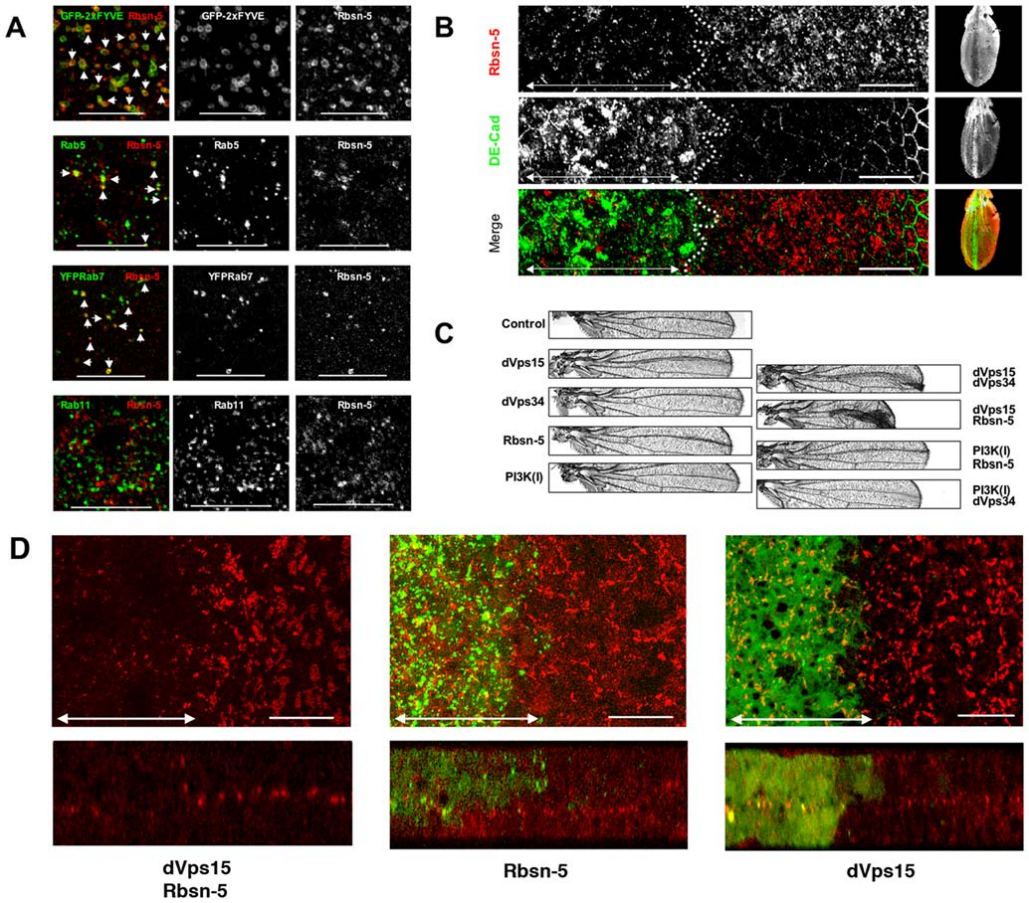
Rbsn-5 is a PI(3)P target protein. (A) Rbsn-5 (red) was co-localized in the pupal wing discs, mainly with GFP-2xFYVE, Rab5 and Rab7 (arrowheads), but not with Rab11. The scale bars represent 20 µm. (B) Rbsn-5 (red) was clearly reduced in the *dVps15*-knockdown region (the left sides of the dotted lines). In the knockdown region, *D*E-Cadherin (green) accumulated intracellularly as shown in Figure 4. (C) Adult wings in which dsRNAs were expressed by *dpp*-Gal4 driver. Malformation was observed near the third vein where the dsRNAs for *dVps15*, *dVps34* or *Rbsn-5* were expressed. This malformation was synergistically enhanced by co-expression of dsRNAs for both *dVps15* and *dVps34* or both *dVps15* and *Rbsn-5*, whereas no such enhancement was observed by co-expression of dsRNAs for both *PI3K class I* and *Rbsn-5* or both *PI3K class I* and *dVps34*. (D) Horizontal (upper) and vertical (lower) sections of the pupal wings expressing dsRNAs for *Rbsn-5* and/or *dVps15*. β-integrin (red) was not localized in the double knockdown region (indicated by double headed arrow in the left panel) whereas marginal defects were observed in the single knockdown (indicated by double headed arrows in the middle and right panels). The right sides of the panels represent the internal control where β-integrin was normally localized to the basal junctions. The scale bars represent 10 µm.

In addition to PI(3)P, Rbsn-5 has been shown to associate with Rab5 and Rab4 and to be controlled by these Rab family small GTPases, which govern endosome fusion and recycling processes, respectively [31]. *Drosophila* Rbsn-5 can bind to the GTP form of Rab5, but not to Rab4 or Rab7 *in vitro* [35], [46]. Although Rbsn-5 has been proposed to function at Rab5 or Rab4-positive endosomes in mammalian cells, we found that it was localized not only at the Rab5-positive endosomes but also the Rab7-positive endosomes, whereas it was absent from Rab11-positive endosomes (Fig. 8A). This supports the notion that Rbsn-5 is involved in the sorting to late endosomes in the degradation pathway.

### Functional interactions between PI3K (III) and Rbsn-5

Finally, we examined the functional relationship between PI3K (III) and Rbsn-5. Although Rbsn-5 contains a FYVE domain that binds to the PI3K (III) product PI(3)P, and it has been shown to be essential for the localization of Rbsn-5 to early endosomes [33], a functional relationship between the two molecules has remained unknown. Firstly, the effect of *dVps15* knockdown on the distribution and abundance of Rbsn-5 was examined. The intensity of Rbsn-5 immunostaining was markedly reduced in the *dVps15* knockdown cells (Fig. 8B). This suggests that PI3K (III) regulates the level of Rbsn-5 protein, in addition to its localization. Next we examined whether Rbsn-5 genetically interacts with PI3K (III). To this end, we examined phenotypes produced by knockdown of *dVps34*, *dVps15*, *Rbsn-5*, or a combination of these (Fig. 8C). dsRNA-mediated knockdown of target mRNAs is usually incomplete, and a low expression level of the mRNA is expected to remain (Fig. 1B). The malformation of wing morphology, a detachment of the dorsal and ventral surfaces, induced by the knockdown of *dVps15* was strongly enhanced by a simultaneous knockdown of *dVps34* or *Rbsn-5* (Fig. 8C). By contrast, we did not find any genetic interaction between *Rbsn-5* or *dVps34* and the *PI3K class I/p110* (Fig. 8C). Since the attachment of the dorsal and ventral wing surfaces requires the basal localization of integrins to form basal junctions, we also examined whether the abnormal localization of β-integrin by the single knockdown for *dVps15* or *Rbsn-5* is enhanced by their double knockdown. To demonstrate the synergistic enhancement, we induced dsRNAs weakly for either *dVps15* or *Rbsn-5* to induce only marginal defects (Fig. 8D). Under this condition, the double knockdown resulted in obvious delocalization of β-integrin (Fig. 8D), which is consistent with the adult wing phenotype. These results support the notion that Rbsn-5 functions under the control of PI3K (III).

## Discussion

In this study, we have demonstrated that PI3K (III) differentially regulates the localization of proteins at distinct membrane domains. The intracellular accumulation of Fmi, *D*E-cadherin and β-integrin induced by the *dVps15* knockdown might be due to defects in the degradation pathway, since the maturation of MVBs and the lysosomal trafficking were defective in these cells. However, unlike these proteins, Fas III did not accumulate in the intracellular compartments, but rather accumulated at the surface of the lateral plasma membrane. It is possible that PI3K (III) regulates proteins at the lateral membrane differently from those localized at other membrane domains. It is also possible that PI3K (III) regulates Fas III in a different way, irrespective of the membrane domain to which it is localized. Whichever is the case it will be important to elucidate the mechanism underlying this difference in a future study.

We have also demonstrated that Rbsn-5, a FYVE domain-containing protein, shares a part of the functions of PI3K (III), in that it is necessary for the regulation of Fas III and β-integrin localization, but not that of *D*E-cadherin and Fmi localization. Although the *Rbsn-5^C241^* null mutant clones may not completely lack Rbsn-5 activity, the requirement of Rbsn-5, or at least the requirement of an appropriate amount, differs between these proteins with respect to normal trafficking. It appears that Rbsn-5 preferentially controls the events at the basolateral regions, given that Rbsn-5 is necessary for the formation of large endosomes at the basal region, whereas it is indispensable for the formation of actin bundles at the apical surface.

PI3K (III) has been implicated in the differential regulation of vesicle trafficking at apical and basolateral regions. For instance, a reduction of PI(3)P dissociates EEA1, a FYVE-domain containing protein essential for early endosome fusion, selectively from basolateral endosomes [15]. However, which proteins, including EEA1, regulate the different trafficking pathways downstream of PI3K (III) has remained unknown. Rbsn-5 has been proposed to be a PI3K (III) effector, since Rbsn-5 harbors a FYVE domain. Our results provide further evidence supporting a possible functional interaction between these two molecules, based on their genetic interaction on the wing morphogenesis and the PI3K (III)-dependent Rbsn-5 immunostaining. Importantly, the different requirement of Rbsn-5 for trafficking at apical junction and basolateral membrane domains suggests that Rbsn-5 may a selective regulator under the control of PI3K (III).

## Materials and Methods

### Fly strains

Flies were reared in vials containing a standard cornmeal medium at 25°C. Conton–S flies were used as the wild type. The transgenic strains used were Tub > YFP Rab7 [47]. Several transgenic strains were expressed by using the Gal4/UAS system [48]. UAS strains were UAS-GFP-Myc-2×FYVE [40] and UAS-GFP-LAMP1 [45], UAS-dVps34 (CG5373) dsRNA, UAS-dVps15 (CG9746) dsRNA, UAS-Rbsn-5 (CG8506) dsRNA; UAS-PI3K Class I (CG4141) dsRNA, UAS-GFP-Rab5 (a gift from K. Matuno), UAS-dVps34 dsRNA; UAS-dVps34 cDNA, UAS-Rbsn-5 cDNA; UAS-Rbsn-5 dsRNA, UAS-dVps34 dsRNA; UAS- dVps15 dsRNA, UAS-dVps34 dsRNA; UAS-Rbsn-5 dsRNA, UAS-PI3K Class I dsRNA; UAS-Rbsn-5 dsRNA, UAS-PI3K Class I dsRNA; UAS-dVps34 dsRNA. UAS-GFP-2xFYVE; UAS-dVps15 dsRNA, UAS-GFP-2xFYVE; UAS-Rbsn-5 dsRNA, UAS-GFP LAMP1; UAS-dVps15 dsRNA, UAS-GFP Rab5; UAS-dVps15 dsRNA, and driver strains were *sd*-GAL4 and *dpp*- GAL4. Fly transformation followed standard methodology.

### Clone generation

Rbsn-5 mutant clones were generated by crossing *y w^−^/Y; P{neoFRT}40A rbn5 c241/Cyo* flies to *y w^−^ hs-FLP; P{w+; Ubi-GFP(S65T)nls}2L P{neoFRT}40A/CyO* flies. Progeny were heat shocked for 2 h at 37.5°C at either the second or third larvae stage. Flies were kept at 25°C up to the white pupal stage and then at 26.5°C until dissection.

### Immunofluorescence microscopy

Staged pupae wings and 3^rd^ larva wing discs were dissected and fixed with 4.0% (w/v) paraformaldehyde (PFA)/PBS solutions for 30 min at room temperature. The fixed wings or discs were incubated with blocking solution (PBS containing 0.2% (w/v) BSA and 0.1% (v/v) Triton X-100) at 4°C for 1 h and then incubated with the following primary antibodies: rat anti-*D*E-cadherin (1∶50 DCAD2 DSHB), mouse anti-Flamingo (1∶20; a gift from T. Uemura), mouse anti-Armadillo (1∶200; N2 7A1 DSHB), mouse anti-Discs large (1∶200; number 4F3 DSHB), mouse anti-FasIII (1∶200; 7G10 DSHB), mouse anti-β-integrin (1∶50;CF.6G11 DSHB), rabbit anti-GFP (1∶300; MBL), rat anti-GFP (1∶300; MBL), rat anti-Rbn5 (1∶300 a gift from T. Tanaka), rabbit anti-Rbn5 (1∶300; a gift from T. Tanaka), mouse anti-120K (1∶200; Calbiochem), rabbit anti-Rab5 (1∶50, a gift from M. Gonzalez-Gaitan), rabbit anti-Rab7 (1∶300; a gift from T. Tanaka), rabbit anti-Rab11 (1∶1000; a gift from S.M. Cohen), rabbit anti-Nuf (1∶3000; a gift from W. Sullivan) and rabbit anti-GM130 (1∶200; Sigma) for 4°C overnight. After the samples were washed with blocking solution five times, the following appropriate secondary antibodies were added and left for 2 h at 4°C; Cy3 conjugated anti-mouse IgG (1∶200; Amersham Pharmacia), Cy3 conjugated anti-rat IgG (1∶200; Amersham Pharmacia), Cy3 conjugated anti-rabbit IgG (1∶200; Amersham Pharmacia), Alexa Fluor 488-conjugated anti-mouse IgG (1∶200; Molecular Probes), Alexa Fluor 488-conjugated anti-rat IgG (1∶200; Molecular Probes), Alexa Fluor 488-conjugated anti-rabbit IgG (1∶200; Molecular Probes) TO-PRO3 (1∶300; Molecular Probes) and Rhodamine phalloidin (1∶300; Molecular Probes). All images were obtained using an Olympus FV-500 confocal microscope and processed using Adobe Photoshop.

### TR-D incorporation assay

Larva wing discs (3^rd^) were dissected and then incubated with 0.5 mM TR-D (MW 3000, Molecular Probes) in Schneider's medium (GIBCO-BRL) containing 10% (v/v) FCS, 50 U/ml penicillin, and 50 mg/ml streptavidin for 5 min at 4°C [Pulse]. After washing twice with ice-cold Schneider's medium, the wing discs were then incubated for 5, 20, 40 and 60 min. with fresh medium at 26.5°C, respectively [Chase]. The wing discs were fixed with 4% (w/v) PFA-PBS solution for 30 min at room temperature and observed using an Olympus FV-500 confocal microscope and processed with Adobe Photoshop.

### Lysotracker staining

Larvae wing discs (3^rd^) were dissected and then stained for 30 sec with Lysotracker red DND-99 (Molecular Probes) diluted 1∶1000 in PBS. Mounting was performed in 80% glycerol in PBS, and the samples were directly visualized by confocal microscopy.

### Electron microscopy

Thirty hour APF staged pupae wings were dissected and fixed with 2.5% (v/v) Glutaldehyde (GA), 2% (w/v) sucrose, 1% (w/v) tannic acid in PBS for 30 min at room temperature and then at 4°C overnight. The samples were then re-dissected to remove the wing cuticles and subjected to post-fixation with 1.0% (v/v) osmium oxide for 1 h on ice. The wings were then dehydrated and embedded in epoxy resin (Epon812, TAAB). Ultrathin sections were obtained using a ultramicrotome (Leica Ultracut UCT), and staining with uranyl acetate and lead citrate. Specimens were observed using a transmission electron microscope (JEOL JEM-1230). For pre-embedding immunoelectron microscopy, dissected pupae were fixed in a mixture of 4.0% (w/v) PFA and 0.01% (v/v) GA in PBS for 30 min at room temperature and then for 1 h at 4°C. The samples were re-dissected with 150 mM (w/v) glycine in PBS to remove the wing cuticles, and incubated with blocking solution (PBS containing 0.1% (w/v) glycine, 5% (v/v) normal goat serum, 0.1% (w/v) saponin, 0.05% (v/v) Triton X-100 and 0.1% (v/v) gelatin) for 30 min at 4°C. Specimens were incubated with a primary antibody solution (PBS containing 1% (v/v) normal goat serum, 0.1% (w/v) saponin, 0.05% (v/v) Triton X-100, 0.1% (v/v) gelatin and rat anti-GFP (1∶10; MBL)) overnight at 4°C and then incubated with anti-rat antibodies conjugated to 1.4 nm gold particles (Nanoprobes) for 3 h at room temperature. The wings were re-fixed with 2% GA in PBS for 15 min at room temperature. They were then treated using an HQsilver kit (Nanoprobes) for 7–10 min at room temperature in the dark. After post-fixing with 0.5% OsO_4_ for 45 min on ice, the wings were dehydrated and embedded into Epon812 resin.
